# Selective Extraction of Flavonoids from *Sophora* *flavescens* Ait. by Mechanochemistry

**DOI:** 10.3390/molecules21080989

**Published:** 2016-07-29

**Authors:** Qihong Zhang, Jingbo Yu, Yingyao Wang, Weike Su

**Affiliations:** 1 National Engineering Research Center for Process Development of Active Pharmaceutical Ingredients, Collaborative Innovation Center of Yangtze River Delta Region Green Pharmaceuticals, Zhejiang University of Technology, Hangzhou 310014, China; zqh1657@126.com (Q.Z.); yjb@zjut.edu.cn (J.Y.); wyy312@126.com (Y.W.); 2 Key Laboratory for Green Pharmaceutical Technologies and Related Equipment of Ministry of Education, College of Pharmaceutical Sciences, Zhejiang University of Technology, Hangzhou 310014, China

**Keywords:** *Sophora flavescens* Ait., flavonoids, mechanochemical-promoted extraction technology, response surface methodology, selectivity

## Abstract

Flavonoids from *Sophora flavescens* were selectively extracted by mechanochemical-promoted extraction technology (MPET) after using response surface methodology to determine the optimal extraction parameters. The highest yield of 35.17 mg/g was achieved by grinding the roots with Na_2_CO_3_ (15%) at 440 rpm/min for 17.0 min and water was used as the sole solvent with a ratio of solvent to solid material of 25 mL/g. Flavonoids prepared by MPET demonstrated relatively higher antioxidant activities in subsequent DPPH and hydroxyl radical scavenging assays. Main constituents in the extracts, including kurarinol, kushenol I/N and kurarinone, were characterized by HPLC-MS/MS, indicating good selective extraction by MPET. Physicochemical property changes of powder during mechanochemical milling were identified by scanning electron microscopy, X-ray powder diffraction, and UV-Vis diffuse-reflectance spectroscopy. Compared with traditional extraction methods, MPET possesses notable advantages of higher selectivity, lower extraction temperature, shorter extraction time, and organic solvent free properties.

## 1. Introduction

*Sophora* *flavescens* Ait. (*S. flavescens*) is a traditional herb medicine distributed in East Asia and some European countries. It has been widely used as a medicine and functional food ingredient for thousands of years because of its potential beneficial properties, such as improving mental health, anti-inflammatory, antiasthmatic, antihelminthic, free radical scavenging and antimicrobial activities [1,2]. Phytochemical research has revealed that the main active compounds in *S. flavescens* are quinolizidine alkaloids and prenylated flavonoids. Chemical and pharmacological research on the quinolizidine alkaloids has been quite thorough [3], whereas study of the flavonoids components remains relatively inadequate. Some research has suggested that flavonoids could enhance immunity, lower blood glucose levels [4], inhibit the activity of tyrosinase enzyme [5] and that they possess anticancer and anti-inflammatory activities [6]. In addition, flavonoids, as phenolic compounds, which usually have significant antioxidant activity, might find potential uses as natural antioxidants, food additives and functional foods. Therefore, the extraction of flavonoids from *S. flavescens* is a worthwhile objective.

Flavonones and flavanonols are the key representative components [7] in *S. flavescens* together with a few isoflavone and flavanone glycosides. However, flavonones and flavanonols are usually poorly water soluble, so normally organic solvents are required to extract flavonoids from *S. flavescens*, using diverse techniques such as conventional heat reflux extraction [8], supercritical fluid extraction [9], microwave-assisted extraction [10] and ultrasound-assisted extraction [11]. Although these methods can obtain mostly active components, heating processes can result in the loss or degradation of target compounds to some extent, and thus increase the cost of production. Moreover, the low selectivity and organic solvent residues make the subsequent purification difficult.

Mechanochemistry is the study of physico-chemical transformations generated by mechanical force, and has been widely applied in mechanical alloying [12], organic synthesis [13], drug modification [14] and degradation of toxic wastes [15]. In recent years, the extraction of natural products from plants is emerging as a new field where mechanochemically promoted reactions can take place with plant materials, such as neutralization reactions between solid reagents and compounds in plant (alkaloids, acids, flavonoids, polyphenols, etc.), formation of soluble glycosides (phytoecdysteroids etc.), and soluble complexes (phytosterols, etc.). MPET has successfully enabled the aqueous extraction of phytoecdysteroids from *Serratula coronate* L. [16], lappaconitine from *Aconitum septentrionale* roots [17], and isofraxidin from *Eleutherococcus senticosus* [18].

Response surface methodology (RSM) is a statistical technique which allows the user to identify optimal conditions for a selected response while minimizing the number of experiments required. This study was designed to develop an environmentally benign and easy to handle method for selectively obtaining flavonoids from *S. flavescens* based on mechanochemical reactions. The mechanical milling and extraction conditions were optimized by applying response surface methodology and the main flavonoids in the extracts were evaluated by high performance liquid chromatography-mass/mass spectrometry (HPLC-MS/MS), which confirmed the good selectivity of the extraction. The antioxidant activities of the extracts were further evaluated to determine the potential for utilization in the food and nutraceutical industries. Scanning electron microscopy, X-ray powder diffraction, and UV-Vis diffuse-reflectance spectroscopy were applied to investigate the physicochemical property changes that occurred during mechanochemical milling.

## 2. Results and Discussion

### 2.1. Preliminary Experiments

#### 2.1.1. Screening of Solid Reagents

**Figure 1:**
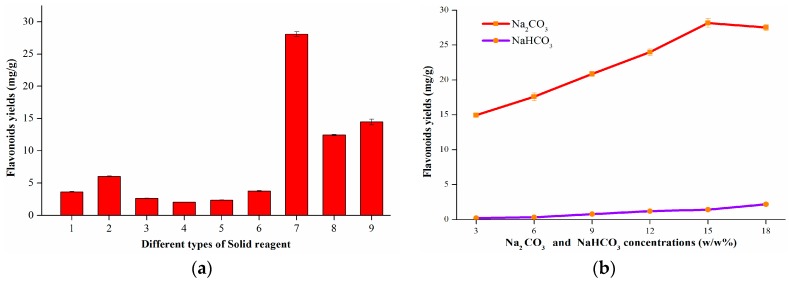
Effects of different solid reagents on flavonoids yield (**a**): (1) blank group: no solid reagent; (2) sodium borate; (3) diatomaceous earth; (4) basic aluminum oxide; (5) Ca_2_CO_3_; (6) NaHCO_3_; (7) Na_2_CO_3_; (8) control group: conventional agitation of unmilled *S. flavescens* powder and unmilled Na_2_CO_3_; (9) control group: conventional agitation of milled *S. flavescens* powder and unmilled Na_2_CO_3_; Effect of Na_2_CO_3_ and NaHCO_3_ concentration on flavonoids yield (**b**). Data are presented as mean ± SD (*n* = 3).

Flavonoids can be potentially transformed into salt forms when ground with alkali. The minimal concentration of solid basic agent required depends on the kinetics of the mechanochemical process (rate of component transfer during mechanochemical treatment) and the content of target compound(s) in the plant tissues. In our preliminary study, several solid reagents were screened (Figure 1a): (1) blank group: no solid reagent; (2) sodium borate; (3) diatomaceous earth; (4) basic aluminum oxide; (5) Ca_2_CO_3_; (6) NaHCO_3_; (7) Na_2_CO_3_; (8) control group: conventional agitation of unmilled *S. flavescens* powder and unmilled Na_2_CO_3_; (9) control group: conventional agitation of milled *S. flavescens* powder and unmilled Na_2_CO_3_. Unexpectedly, diatomaceous earth, basic aluminum oxide, and Ca_2_CO_3_ had no positive effect on the yield of flavonoids, giving even lower yields than the blank group. It was proposed that the adsorbability of diatomaceous earth and basic aluminum oxide might give rise to these decreased yields. The pK_b_ values of Na_2_CO_3_, sodium borate, Ca_2_CO_3_, and NaHCO_3_ are 3.67, 4.76, 5.0, 7.65, respectively, which indicated that stronger alkalinity of the solid basic agents resulted in better extraction yield of the flavonoids. However, the pK_b_ value alone cannot explain why Ca_2_CO_3_ decreased the yield to give a yield that was lower than that of the blank group. One possible reason might be its poor solubility in water. In terms of food safety, sodium borate was discarded. It could be see that the milled group (7) had a better yield than the control groups (8) and (9), which indicated that milling could increase the yield, and milling with a solid reagent could increase the yield better than only milling the plant material followed by adding Na_2_CO_3_. Na_2_CO_3_ and NaHCO_3_ were chosen for further investigation at different concentrations of 3.0%, 6.0%, 9.0%, 12.0%, 15.0% and 18.0% respectively (wt %, mass ratio of Na_2_CO_3_ to *S. flavescens* particles) under the same extraction conditions (grinding time: 10 min; rotational speed: 400 rpm; extraction solvent: water; extraction time: 20 min; ratio of solvent to material: 30 mL/g; acidification pH: 5.0; extraction temperature: 25 °C). By comparing the results (Figure 1b), it was clear that Na_2_CO_3_ could achieve more than ten times better yield than NaHCO_3_. Therefore, Na_2_CO_3_ was applied as the optimal solid reagent.

#### 2.1.2. Acidification pH

**Figure 2:**
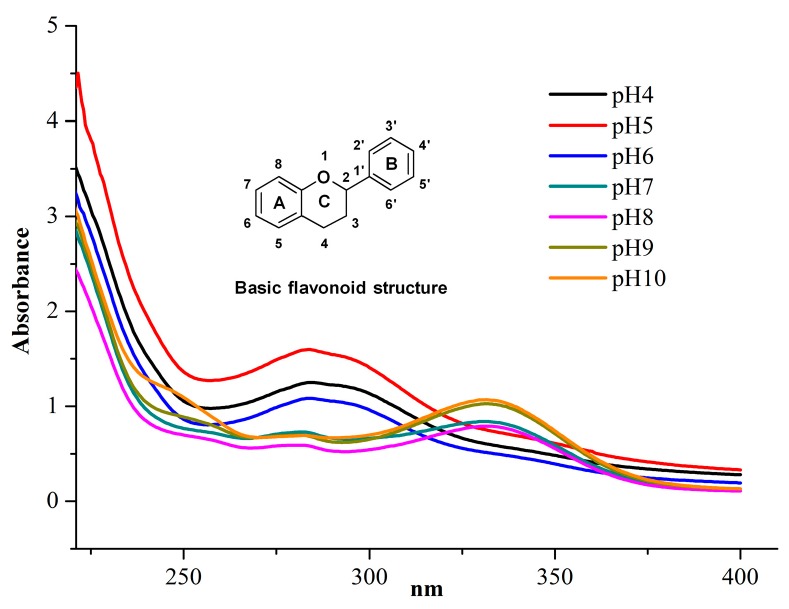
UV-Vis absorption spectra.

As depicted in Figure 2, acidification pH values from 4–6 exhibited the maximum absorption wavelengths at about 291.00 nm and a weak shoulder absorption wavelength at about 339.00 nm, which correspond to the intense absorptions of flavonones and flavanonols. The main absorption was induced by the benzoyl system, and the weak shoulder peak was due to the B-ring of the flavonoid molecule that could not conjugate with the carbonyl group in the pyran ring. However, as the acidification pH ranged from 7 to 10, the maximum absorption wavelength was seen at 329.98 nm as well as a weak absorption band at 282.44 nm, which suggested that *o*-phenolic hydroxy groups were substituted [19]. Moreover, under mechanochemical treatment, in addition to the 7- or 4’-OH on flavonoid molecules that could react with Na_2_CO_3_, the 3-OH could be neutralized by Na_2_CO_3_ with a corresponding shift of the absorption wavelength from a long wavelength (free state) to a shorter wavelength (substituted state) [19]. This was identical to the curve seen at pH 7–10, even though it could not be carried out under normal conditions. Because flavonoids could be completely transformed into their precursors at pH 6, the optimal acidification pH value of 6 was selected.

### 2.2. Optimization of the Operating Parameters

Preliminary experiments to determine the main factors and the appropriate ranges of the CCD were performed. The range of rotational speed, milling time, solid reagent amount and solvent to material ratio were determined based on preliminary single factor experiments.

#### 2.2.1. Model Fitting

The values of responses (yield of flavonoids) under different experimental combinations are given in Table 1. The significance of each coefficient was determined using the *p*-value (Table 2). The corresponding variables would be more significant if the *p*-value becomes smaller. It was found that the variables rotational speed (X_1_), grinding time (X_2_), solvent to material ratio (X_3_), amount of solid reagents (X_4_) and their quadratic parameters were highly significant at the level of *p* < 0.01. The interactions of X_1_X_2_ and X_3_X_4_ were highly significant (*p* < 0.01), and X_2_X_3_ were significant (*p* < 0.05). The regression model can be described by the following quadratic polynomial:

*Y* = − 53.98156 + 0.21282X_1_ + 2.11663X_2_ + 1.05188X_3_ + 1.41674X_4_ + 0.000848750X_1_X_2_ + 0.000256250X_1_X_3_ − 0.000356250X_1_X_4_ − 0.00717500X_2_X_3_ − 0.00379167X_2_X_4_ + 0.018625X_3_X_4_ − 0.000259906X_1_^2^ − 0.065412X_2_^2^ − 0.025462X_3_^2^ − 0.055451X_4_^2^ (1)

#### 2.2.2. Analysis of Variance

The coefficients of the above Equation (1) were calculated, and the linearity and quadratic effect of the treatment variables, their interactions and coefficients on the response variables were obtained by analysis of variance (ANOVA, Table 2). For each term in the model, a small *p*-value (*p* < 0.05) and a large *F*-value would imply a more significant effect on the extraction yield [20]. The linear coefficients (X_1_, X_2_, X_3_ and X_4_), quadratic term coefficients (X_1_X_1_, X_2_X_2_, X_3_X_3_ and X_4_X_4_) and interaction coefficients (X_1_X_2_, X_2_X_3_, and X_3_X_4_) were significant, with very small *p*-values (*p* < 0.05). The determination coefficient (R^2^ = 0.9962) of the quadratic regression model indicated that only 0.38% of the total variations were not explained by the model. The value of the adjusted determination coefficient (Adj-R^2^ = 0.9926) also confirmed that the model was highly significant, which indicated good agreement between the experimental and predicted values of flavonoid yield. The analysis of error results indicated that the lack of fit test (0.1050) was insignificant at the 95% confidence level, confirming the validity of the model. Moreover, the model *p*-value (Prob > *F*) was very low (<0.00001), indicating that the model terms were significant.

#### 2.2.3. Analysis of Response Surfaces and Optimal Processing Conditions

**Figure 3:**
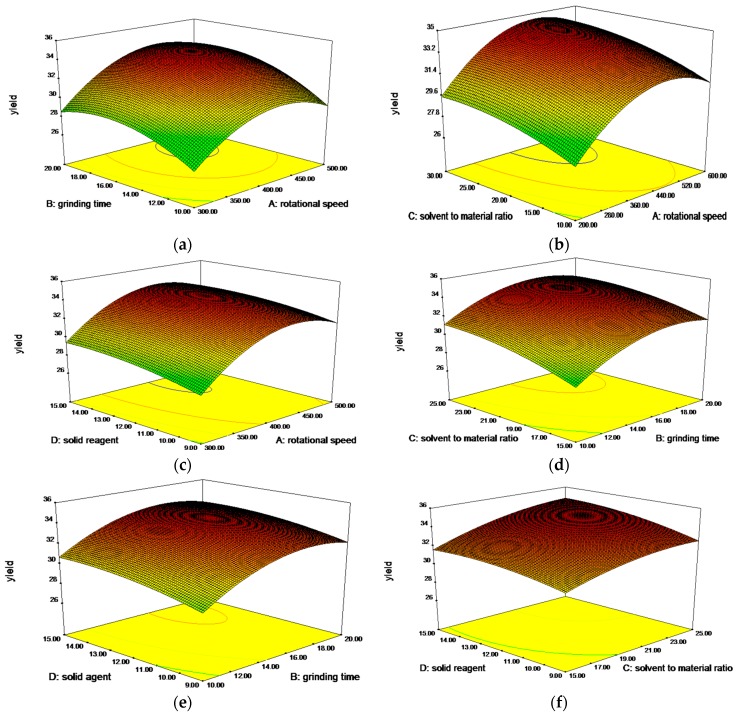
Response surface (3D) showing the effect of variables on total flavonoid extraction. (**a**) rotational speed and grinding time; (**b**) solvent to material ratio and rotational speed; (**c**) solid reagents and rotational speed; (**d**) solvent to material ratio and grinding time; (**e**) solid reagents and grinding time; (**f**) solvent to material ratio and solid reagents.

Through the 3D plots and their respective contour plots, it was very easy to understand the interactions between two variables and to determine their optimum levels (Figure 3). Figure 3a,f shows the 3D graphic surface and contour plot of the combined effects of rotational speed and grinding time (X_1_X_2_), and ratio of solvent to material and amount of solid reagent (X_3_X_4_). The tortuous surface and oval contour plot show a very strong interaction between the factors (X_1_ and X_2_, X_3_ and X_4_, *p* < 0.01). The effects of grinding time and ratio of solvent to material on extraction yield are shown in Figure 3d, where interaction between the two factors was also strong (*p* < 0.05). As shown in Figure 3b,c,e, the interactions between rotational speed and ratio of solvent to material (X_1_X_3_), rotational speed and amount of solid reagent (X_1_X_4_), grinding time and amount of solid reagent (X_2_X_4_) were not significant. On the basis of the response surfaces, the optimal conditions were determined as follows: rotational speed of 439.74 rpm, grinding time of 17.22 min, ratio of solvent to material of 25:1, amount of solid reagent of 14.97%. Under the optimal conditions, the model gave a maximum predicted value of 35.36 mg/g. For operational convenience, the optimal extraction parameters were set as a rotational speed of 440 rpm, grinding time of 17 min, ratio of solvent to material of 25:1 and amount of solid reagent of 15%. Triplicate experiments were performed under the determined conditions and they yielded 35.17 mg/g, in agreement with the predicted value, indicating that the model was adequate for the extraction process.

### 2.3. The Selectivity Analysis by HPLC-MS/MS

**Figure 4:**
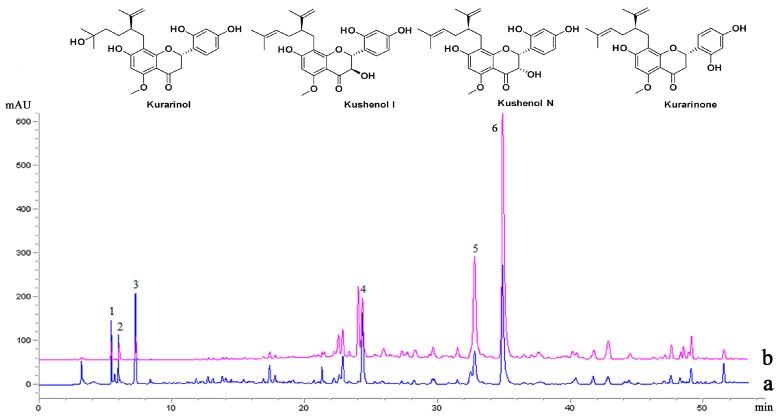
HPLC for different extraction methods: (**a**) conventional heating extraction; (**b**) mechanochemical-promoted extraction technology.

In Figure 4 lines a–b correspond to the conventional heating extraction (CHE) and MPET extracts, respectively. The peaks appearing in b were essentially identical to those in a, suggesting that MPET could extract the same kinds of flavonoids as CHE. However, some differences were observed. The peak-area gaps between 4, 5, 6 and 1, 2, 3 in trace b were bigger than that in a. To investigate the mechanism that caused this peak-area gap, peaks 4, 5, 6 were identified by comparing their UV absorbance and MS data based on some references [21,22]. The UV λ_max_ of peaks 4, 5, 6 had the characteristic absorptions of flanonoids at 230 nm, 285 nm, and 330 nm (s), and their [M + H]^+^ ions were selected for collision-induced dissociation (CID) fragmentation to produce MS/MS spectra (See Supplementary Materials), which allowed their identification as kurarinol, kushenol I/N, and kurarinone, respectively, while peaks 1, 2, 3 had relatively low UV absorptions at 220 nm, 209 nm and 223 nm, and were determined them to be alkaloids according to the typical UV and MS data of alkaloids [23]. This indicated that MPET could selectively extract the flavonoid components rather than alkaloids.

### 2.4. Antioxidant Activities in Vitro

#### 2.4.1. The DPPH Radical Scavenging Activity

**Figure 5:**
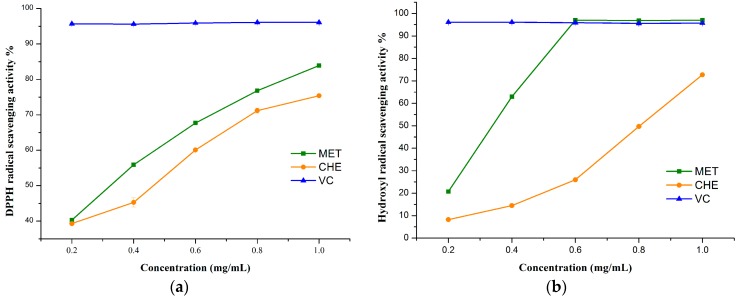
Antioxidant activities of flavonoids. (**a**) DPPH radical scavenging activity; (**b**) hydroxyl radical scavenging activity. Data were presented as mean ± SD (*n* = 3).

Scavenging of DPPH radicals is a common antioxidant assay used to determine the antioxidant activities of compounds. As shown in Figure 5a, the flavonoids prepared by MPET and CHE showed concentration dependent radical scavenging effects, although they were weaker than those of vitamin C (Vc) in the same concentration. Apparently, flavonoids obtained by MPET displayed relatively higher antioxidant activity than those obtained by CHE. It was supposed that MPET had better selectivity for the extraction of flavonoids, resulting in higher purity, which is better for the antioxidant activities.

#### 2.4.2. Hydroxyl Radical Scavenging Activity

Hydroxyl radical, which is well known as one of the most reactive free radicals, can react with almost all biomacromolecules in living cells and induce severe damages. As shown in Figure 5b, flavonoids obtained by MPET displayed relatively higher antioxidant activity than that of CHE. At the concentration of 0.6 mg/mL, the scavenging ability of MPET was 97.01%, which was slightly better than Vc (95.7%), so the flavonoids from *S. flavescens* might be good antioxidants for the functional food industries.

### 2.5. Physicochemical Property Changes during Mechanochemical Milling

#### 2.5.1. Morphology Analysis

**Figure 6:**
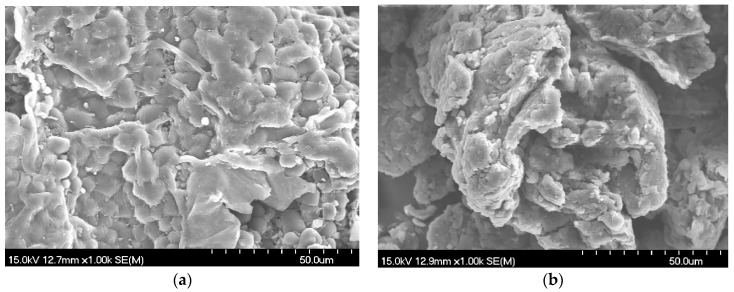
SEM micrograph of: (**a**) physical mixture; (**b**) mechanochemical mixture.

Morphology changes were observed by scanning electron microscopy (SEM). It could be clearly seen that the shattered powder (Figure 6a) had some intact tissues and closed cells. However, powders after grinding treatment (Figure 6b) had a relatively homogeneous small particle size. Integration with cracks on the particle surface and agglomeration phenomena occurred. Mechanical force could not only result in a diminishing of particle sizes, and breaking of cell walls, but also lead to some physicochemical changes, such as electric charge changes on the particle surface by the strong squeezing and shearing forces, which facilitated the release of substances within the cell.

#### 2.5.2. XRD Analysis

**Figure 7:**
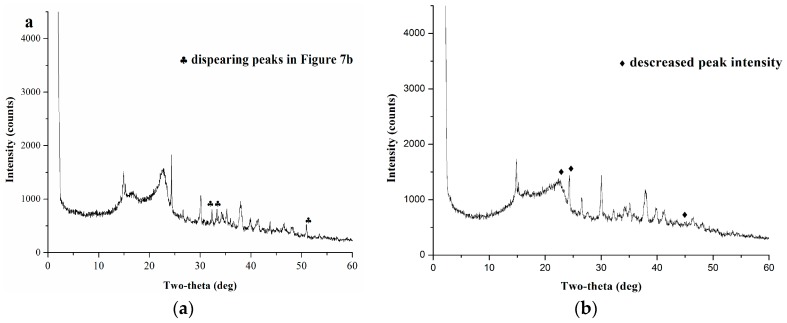
X-ray diffraction spectra of (**a**) physical mixture; (**b**) mechanochemical mixture.

The XRD diagrams of a physical mixture and a mechanochemical mixture of *S. flavescens* powder with Na_2_CO_3_ are shown in Figure 7a,b. The physical mixture exhibited several sharp 2θ peaks at 14.826°, 22.706°, 24.128°, 30.053°, 37.874°, which were assigned to the crystalline structure of compounds contained in *S. flavescens* (Figure 7a). However, the mechanochemical mixture behaved as a semi-crystalline material, showing a considerable decrease in the intensities of the main 2θ peaks at 22.512°, 24.307°, 43.742°, and some peaks of medium intensity at about 15°–22°of 2θ with underlaying scattering, due to some amorphous contents (Figure 7b). Some 2θ peaks at 33.298°, 33.583°, 50.913° disappeared while new peaks appeared at 26.437°, while some 2θ peaks at 14.775°, 29.986°, 37.797°, 39.741°, 41.269° and 46.270° showed increased intensities (Figure 7b). As to the crystalline portion, the main reflections of Na_2_CO_3_ were also present at 25.923°, 27.108°, 30.070°, 32.884°, 34.217°, 34.365°, 35.106°, 37.920°, 38.512°, 39.697°, 40.882°, 44.436°, 46.065°, 47.102°, 48.139°, 53.417°, 54.655° of 2θ, and the diffractogram of the mechanochemical mixture showed a little backward shift. All of the above indicated substantial transformations into highly active phase(s) with low crystallinity which properties were different from those of the physical mixture. Therefore, mechanochemical pretreatment might result in a notable amorphization and the possible formation of flavonoids-base salts. The above results demonstrated that mechanochemical pretreatment would help the transformation of the chemical substances’ status, and might promote the neutralization reaction between flavonoids and the solid basic reagents.

#### 2.5.3. UV-Vis Diffuse-Reflectance Analysis

**Figure 8:**
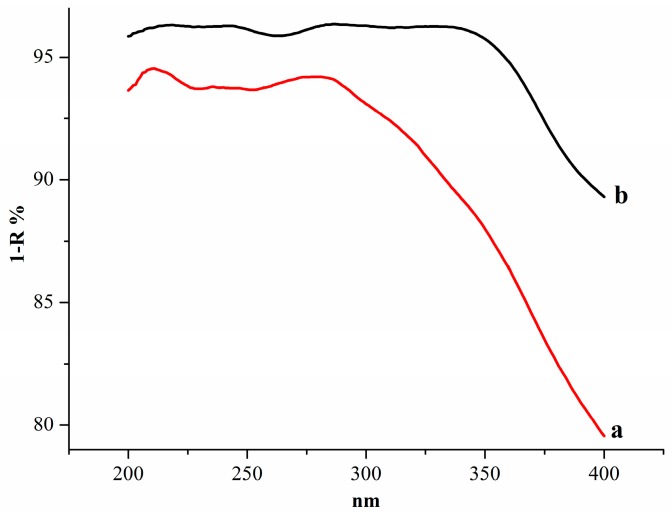
UV-Vis diffuse-reflectance spectra of: (**a**) physical mixture; (**b**) mechanochemical mixture.

As shown in Figure 8, lines a, b show the UV-Vis diffuse-reflectance spectra of the physical mixture and mechanochemical mixture, respectively. Compared to a, curve b had more absorption and a slight red shift, suggesting that neutralization reactions in the solid state might occur, which was consistent with Figure 2. It is well known that mechanical treatment could crack the plant cell walls, distort its tissues, and lead to the reactions between solid reagents and active compounds, which would change the surface properties of the plant powders and give rise to the different reflectivity.

### 2.6. Comparison with Different Methods in Flavonoids Extraction

Table 3 summarizes an overall comparison of the characteristics of different extraction methods. MPET gave the highest flavonoids content of 4.76% from *S. flavescens*. However, poor selectivity can be determined from the lower contents of 1.98%, 1.73%, and 2.01% seen in the traditional extraction process, no matter whether ultrasonic or microwave techniques, or conventional heating were applied. MPET could extract flavonoids using water at 25 °C, while conventional heating, ultrasonic or microwave techniques needed to use different ethanol/water cosolvents to extract flavonoids at higher temperature. Furthermore, MPET also displayed obviously advantages over ultrasonic treatment and conventional heating method in both extraction time and flavonoid yields. With the extraction time of 29.5 min, the ultrasonic technique could obtain a yield of 33.56 mg/g, and CHE could only get a yield of 33.87 mg/g with an extended extraction time of 120 min, which was time consuming. Although a slightly higher yield was achieved by the microwave method, 60% ethanol was required as extraction solvent at an elevated microwave power setting power (420 W), which gave rise to higher cost and energy consumption. It was thus proposed that the shear force and instant high pressure during mechanochemical treatment that caused the breakage of cell walls, lowered diffusion hindrances and finally initiated mechanochemical reactions to afford soluble salts of the target compounds, efficiently facilitated the extraction process and increased the extraction efficiency and yield.

## 3. Materials and Methods

### 3.1. Materials and Reagents

*S. flavescens* roots were purchased from Zhejiang CONBA Pharmaceutical Co., Ltd. (Hangzhou, China) and shattered by a HC-500T2 pulverizer (Song Qing Hardware Factory, Yongkang, China) to an average particle size of 0.5 mm. The powder was then stored in a dry place at room temperature. Standard rutin, analytical grade reagents sodium carbonate and citric acid were purchased from Sinopharm Chemical Reagent Co., Ltd. (Shanghai, China). Analytical-grade reagents were purchased from Tianjin Yongda Chemical Reagent Development Centre (Tianjin, China) and HPLC grade solvents were purchased from Tedia Company Inc. (Fairfield, CA, USA).

### 3.2. Mechanochemical-Promoted Extraction Technology (MPET)

In z preliminary study, various kinds of solid reagents such as sodium borate, diatomaceous earth, basic aluminum oxide, Ca_2_CO_3_, Na_2_CO_3_ and NaHCO_3_ were screened at an excess dosage of 25% (wt %, mass ratio of solid reagents to *S. flavescens* particles). The extraction procedure was as follows: *S. flavescens* roots (10.0 g), solid reagents, and 72 g of stainless steel balls with 12 mm diameter were added into a 50 mL vial (PM-200 planetary mill, Retsch, Haan, Germany). After co-grinding at 400 rpm for 10 min, the powders were extracted with water for 20 min and then centrifuged at 3077 g for 10 min. The solution pH was adjusted to 4–5 with citric acid. The solution was condensed, centrifuged at 9391 *g*, the supernatant was discarded and the precipitants were analyzed by ultraviolet spectrophotometry (UV-2550 PC, Shimadzu, Kyoto, Japan) and HPLC/MS (Agilent 1100 HPLC system, Santa Clara, CA, USA) consisting of a Surveyor autosampler, pumps and photodiode array detector connected to an LCQ-Advantage ion trap mass spectrometer (Thermo Finnigan, San Francisco, CA, USA) fitted with an ESI source. Acidification pH value was optimized by acidifying the extracted solution to the pH values of 10 to 4, and then analyzing the extracts.

### 3.3. Conventional Heating Extraction (CHE)

According to the optimal conditions, *S. flavescens* roots (10.0 g) were refluxed with ethanol-water (200 mL, 80:20, *v*/*v*) solution at 85 °C for 1 h, and then the mixture was centrifuged for 10 min at 3077 *g*. The process was repeated two times, the supernatants were combined, condensed and analyzed by ultraviolet spectrophotometry and HPLC.

### 3.4. Experimental Design

RSM was used to determine the optimal MPET conditions for the extraction of flavonoids from *S. flavescens* roots. To explore the effect of independent variables on the response within the range of investigation, a central composite rotate design with four independent variables (X_1_, rotational speed; X_2_, grinding time; X_3_, solvent to material ratio and X_4_, amount of solid reagents) at five levels [24] was performed with some modifications, as shown in Table 4. The variables were coded according to Equation (1): x = ($X_{i}$ – ${\text{X}}_{0}$)/Δ$X_{i}$, where Xi is the coded value of an independent variable, X_i_ is the real value of the independent variable, X_0_ is the real value of an independent variable at the centre point, and ΔX_i_ is the step change value.

The yield of flavonoids was considered as the dependent variable or response. For a central composite rotate design with four independent variables at five levels, 30 experimental runs are required. The actual design of experiments is given in Table 2. The experimental results were fitted to a second-order polynomial model, and the regression coefficients were determined. The quadratic model for predicting the optimal point was expressed according to Equation: Y = $\beta_{0}+\overset{4}{\underset{i=1}{\sum }}\beta_{i}X_{i}+\overset{4}{\underset{i=1}{\sum }}\beta_{\text{ii}}X_{i}{}^{2}\;+\overset{4}{\underset{j=1}{\sum }}\beta_{\text{ij}}X_{i}X_{j},$ where β_0_, β_i_, β_ii_ and β_ij_ are constant regression coefficients of the model, while *X_i_*, *X_j_* are the independent variables.

### 3.5. Total Flavonoids Content

The total flavonoid content from *S. flavescens* in extracts was determined according to the NaNO_2_-Al(NO_3_)_3_-NaOH colorimetry with some modifications [25]. The reaction mixture contained 2.0 mL of extract, 6 mL of 60% ethanol, and 1 mL of 5% sodium nitrite. Six minutes later, 1 mL of 10% aluminum nitrite was added. In the next six minutes, 10 mL of 1 M sodium hydroxide solution were added and the volume was adjusted to 25 mL by adding 60% ethanol. Immediately, the reaction mixture absorbance was measured by UV spectrophotometry at 510 nm against a blank (control) and used to calculate yields using rutin as a standard. Measurements were calibrated to a standard curve y = 3.239x − 0.020, (R^2^ = 0.999). Flavonoid content is calculated as follows:

Yield = flavonoid content of extracts (mg)/weight of *S. flavescens* powder (g) × 100%

### 3.6. HPLC-MS Analysis

The chromatographic separation was performed on XB-C_18_ column (4.6 mm × 250 mm, 5 μm, Welch, Shanghai, China) at 30 °C. Solvent A (formic acid) and solvent B (acetonitrile) were selected as the mobile phases at the flow rate of 1.0 mL/min. The linear gradient elution started with 0 min, 90% A; 20 min, A 60%; 40 min, 40% A; 60 min, 5% A. The signal was monitored at 280 nm using the diode array detector. MS analysis was carried out in the positive ion mode recorded over a mass range of 120–500 *m*/*z*. Nitrogen (N_2_) was used as the sheath and auxiliary gas, and helium (He) was used as the collision gas. Capillary voltage was 24 V, capillary temperature was 300 °C, and sheath gas flow rate was 40 mL/min.

### 3.7. Antioxidant Activities

#### 3.7.1. Assay of DPPH Radical Scavenging Activity

The scavenging effect of the flavonoids extracted by MPET and CHE on DPPH radical was compared basing on Brand-Williams et al. [26] with some modifications. Briefly, 2.0 mL of 0.2 mM DPPH in anhydrous ethanol was added to 2.0 mL of sample (at different concentrations). The mixture was shaken and incubated for 30 min at room temperature in dark, and the absorbance of the resulting solution was measured at 517 nm. Ascorbic acid was used as control substance. The scavenging percentage was calculated according to the following equation:

Scavenging percentage % = (A_0_ + A_1_ − A_2_)/A_0_ × 100%

where A_2_ was the absorbance of the test sample; A_0_ was the absorbance of the control group; and A_1_ was the absorbance of 2.0 mL sample in 2.0 mL anhydrous ethanol.

#### 3.7.2. Assay of Hydroxyl Radical Scavenging Activity

Hydroxyl radical scavenging activity was determined according to the literature with some modifications [27]. One mL sample solutions of different concentration, 0.5 mL of salicylic acid-ethanol solution (9.0 mM), 0.5 mL of FeSO_4_ solution (9.0 mM) and 3.0 mL of distilled water were successively mixed in a tube. The reaction was initiated by the addition of 2.0 mL H_2_O_2_ (8.8 mM) to the mixture above, and the absorbance at 510 nm was recorded. The hydroxyl radical scavenging activity was calculated as follows:

Scavenging percentage % = (A_0_ + A_1_ − A_2_)/A_0_ × 100%

where A_2_ was the absorbance of the test sample; A_0_ was the absorbance of the control group (deionized water instead of sample); and A_1_ was the absorbance of water instead of H_2_O_2_.

### 3.8. Physicochemical Property Analysis

The morphology of the samples with different pretreatments was observed using scanning electron microscopy (S-4700, Hitachi, Tokyo, Japan). The X-ray powder diffraction (XRD) analysis was carried out on a ARL X'TRA X-ray diffractometer (Thermo, Waltham, MA, USA). Diffuse-reflectance UV-Vis spectra were acquired with the Shimadzu UV-2600 spectrometer (Shimadzu).

### 3.9. Statistical Analysis

The results were expressed as means of yield ± SD (standard deviation). Design-Expert 8.0.6 (Stat-Ease Inc., Minneapolis, MN, USA) was used to calculate the coefficients of the quadratic polynomial model and the optimization. *p* values of less than 0.05 were considered to be statistically significant.

## 4. Conclusions

The present study showed that MPET is a high efficiency and green method to selectively extract flavonoids from *S. flavescens*. The extraction variables were optimized through RSM, whereby a rotational speed of 440 rpm, grinding time of 17 min, solvent to material ratio of 25 mL/g and amount of solid reagents of 15% were proved to be the best conditions to maximize the total yield of flavonoids. With regard to antioxidant activity results, a higher radical scavenging activity was obtained for the flavonoids extracts obtained by MPET than with CHE, which suggested that flavonoids from *S. flavescens* might be used as a source of natural antioxidants in the functional food industry. It is also promising that MPET can conveniently be applied in the rapid manufacture of other natural products, especially for poorly water-soluble components.

## Figures and Tables

**Table 1:** The central composite experimental design and their response.

Experiment Number	Coded Variables				Actual Variables				Yield (mg/g)
	$x_{1}$	$x_{2}$	$x_{3}$	$x_{4}$	$X_{1}$	$X_{2}$	$X_{3}$	$X_{4}$	
1	−1	−1	−1	−1	300	10	15	10	23.62
2	1	−1	−1	−1	500	10	15	10	25.65
3	−1	1	−1	−1	300	20	15	10	25.95
4	1	1	−1	−1	500	20	15	10	30.44
5	−1	−1	1	−1	300	10	25	10	24.99
6	1	−1	1	−1	500	10	25	10	28.76
7	−1	1	1	−1	300	20	25	10	27.13
8	1	1	1	−1	500	20	25	10	32.08
9	−1	−1	−1	1	300	10	15	20	24.31
10	1	−1	−1	1	500	10	15	20	27.17
11	−1	1	−1	1	300	20	15	20	26.97
12	1	1	−1	1	500	20	15	20	30.95
13	−1	−1	1	1	300	10	25	20	27.82
14	1	−1	1	1	500	10	25	20	30.15
15	−1	1	1	1	300	20	25	20	29.42
16	1	1	1	1	500	20	25	20	33.78
17	−2	0	0	0	200	15	20	15	19.41
18	2	0	0	0	600	15	20	15	27.20
19	0	−2	0	0	400	5	20	15	24.08
20	0	2	0	0	400	25	20	15	30.24
21	0	0	−2	0	400	15	10	15	28.37
22	0	0	2	0	400	15	30	15	33.94
23	0	0	0	−2	400	15	20	5	30.03
24	0	0	0	2	400	15	20	25	33.38
25	0	0	0	0	400	15	20	15	33.15
26	0	0	0	0	400	15	20	15	33.63
27	0	0	0	0	400	15	20	15	33.45
28	0	0	0	0	400	15	20	15	33.56
29	0	0	0	0	400	15	20	15	33.74
30	0	0	0	0	400	15	20	15	33.65

**Table 2:** Results of the ANOVA to the response surface quadratic model.

Source	df	Sum of Squares	Mean Square	*F*-Value	*p*-Value
Model	14	425.74	30.41	279.18	<0.0001 **
$X_{1}$	1	81.96	81.96	752.39	<0.000 1**
$X_{2}$	1	55.72	55.72	511.57	<0.0001 **
$X_{3}$	1	38.03	38.03	349.11	<0.0001 **
$X_{4}$	1	14.49	14.49	133.05	<0.0001 **
$X_{1}X_{2}$	1	2.88	2.88	26.45	0.0001 **
$X_{1}X_{3}$	1	0.26	0.26	2.41	0.1413
$X_{1}X_{4}$	1	0.18	0.18	1.68	0.2148
$X_{2}X_{3}$	1	0.51	0.51	4.73	0.0461 *
$X_{2}X_{4}$	1	0.052	0.052	0.48	0.5012
$X_{3}X_{4}$	1	1.25	1.25	11.46	0.0041 **
$X_{1}{}^{2}$	1	185.28	185.28	1701.00	<0.0001 **
$X_{2}{}^{2}$	1	73.35	73.35	673.40	<0.0001 **
$X_{3}{}^{2}$	1	11.11	11.11	102.04	<0.0001 **
$X_{4}{}^{2}$	1	6.83	6.83	62.72	<0.0001 **
Residual	15	1.63	0.11		
Lack od fit	10	1.41	0.14	3.21	0.1050
Pure error	5	0.22	0.044		
Cor total	29	427.37			
R^2^ = 0.9962					
Adj-R^2^ = 0.9926					

Note: * *p* < 0.05 significant, ** *p* < 0.01 highly significant.

**Table 3:** Comparison of different flavonoid extraction methods.

Method	Extraction Solvent	Extraction Temperature (°C)	Extraction Time (min)	Content (%)	Extraction Yield (mg/g)
MPET	water	25	20	4.76 ± 0.14	35.17 ± 0.06
Ultrasound	44.20% ethanol	57	29.5	1.98 ± 0.09	33.56 ± 0.11
Microwave	60% ethanol	- ^a^	25	1.73 ± 0.08	35.35 ± 0.09
Conventional heating	80% ethanol	85	60 + 60	2.01 ± 0.13	33.87 ± 0.10

Data are presented as mean ± SD (*n* = 3). ^a^ microwave power 420 W.

**Table 4:** Comparison of results of MPET and CHE.

Independent Variables	Symbol	Coded Factor Level				
		−2	−1	0	1	2
Rotational speed (rpm)	X_1_	200	300	400	500	600
Grinding time (min)	X_2_	5	10	15	20	25
Solvent to material ratio	X_3_	10:1	15:1	20:1	25:1	30:1
Solid reagents (%)	X_4_	6	9	12	15	18

## Supplementary Materials

Supplementary materials can be accessed at: http://www.mdpi.com/1420-3049/21/8/989/s1.

## Abbreviations

The following abbreviations are used in this manuscript:

*Sophora flavescens*	*S. flavescens*
MPET	Mechanochemical-promoted extraction technology
CHE	Conventional heating extraction
RSM	Response surface methodology
