# Supplementary material for: Selective Extraction of Flavonoids from Sophora flavescens Ait. by Mechanochemistry
Source: Molecules. 2016 Jul 29;21(8):989. doi: 10.3390/molecules21080989 (PMC6273523; doi:10.3390/molecules21080989)
Supplement: Supplementary file 1 [file molecules-21-00989-s001.pdf]

Supplementary Materials: Selective Extraction of Flavonoids from *Sophora flavescens* Ait. by Mechanochemistry

Qihong Zhang, Jingbo Yu, Yingyao Wang and Weike Su

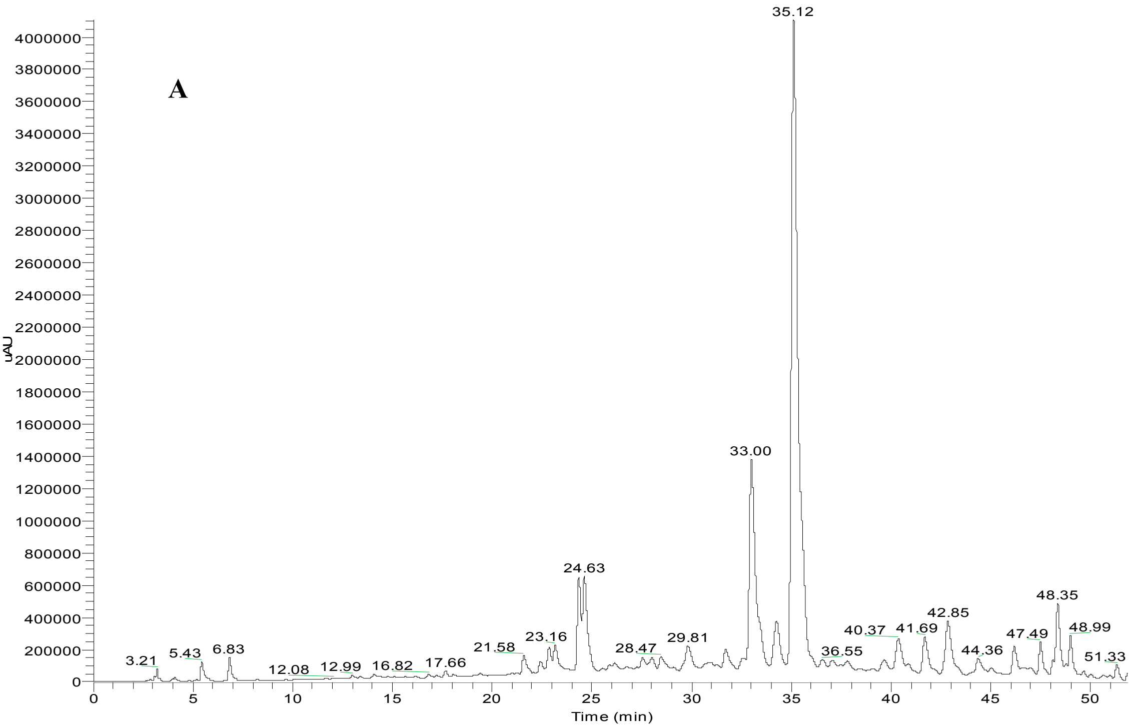

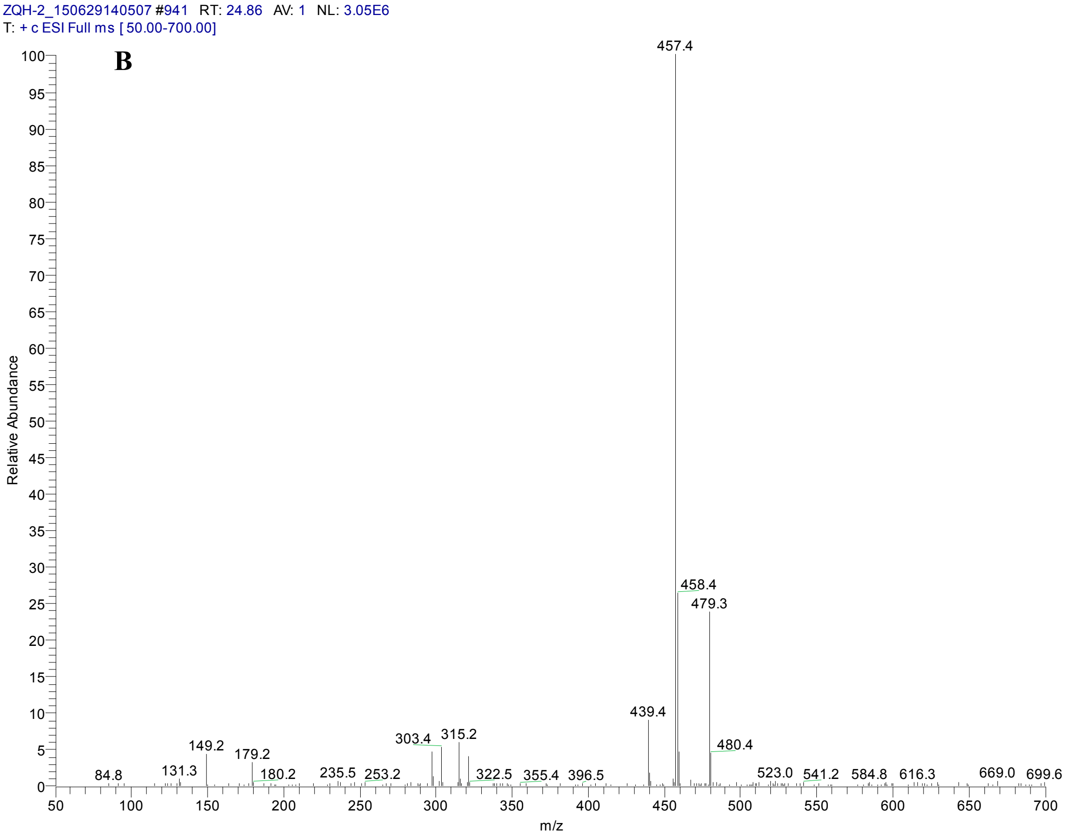

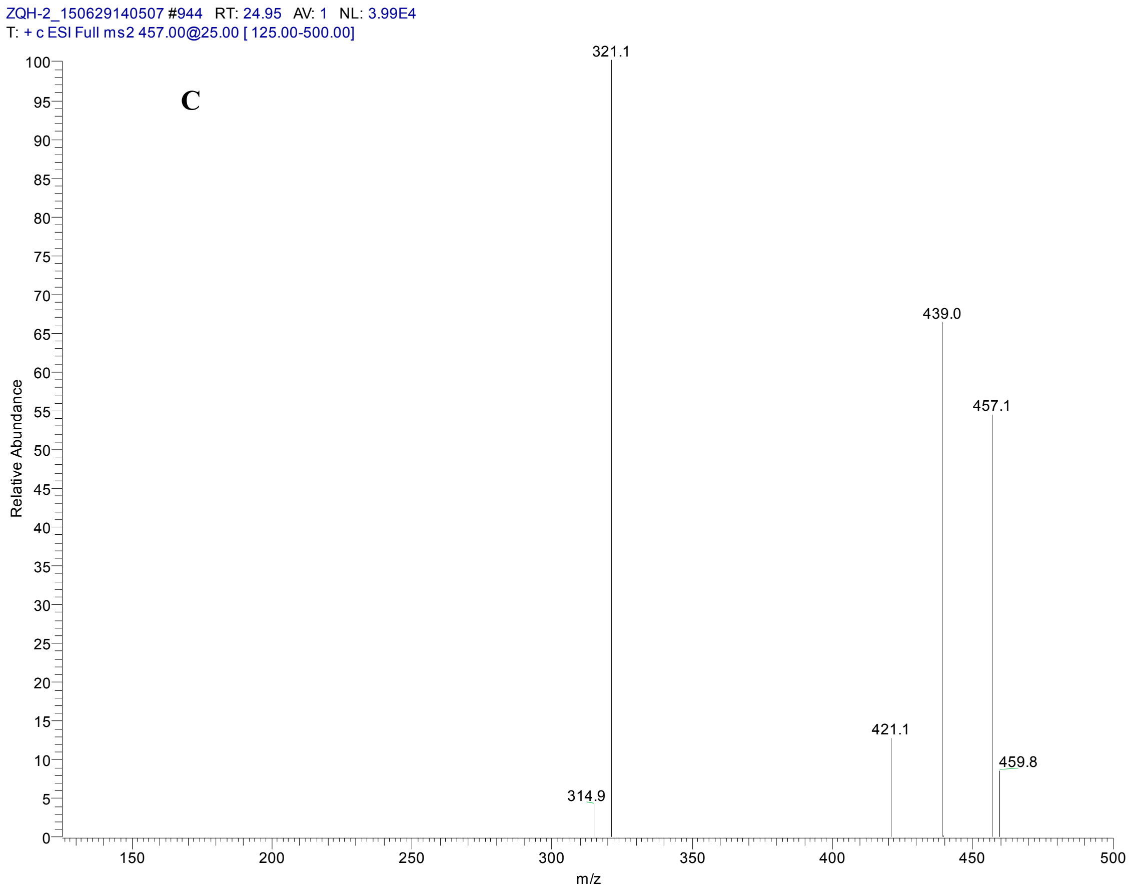
ZQH-2_150629140507 #944 RT: 24.95 AV: 1 NL: 3.99E4
T: + c ESI Full ms2 457.00@25.00 [125.00-500.00]
C
Relative Abundance
m/z
321.1
439.0
457.1
421.1
459.8
314.9

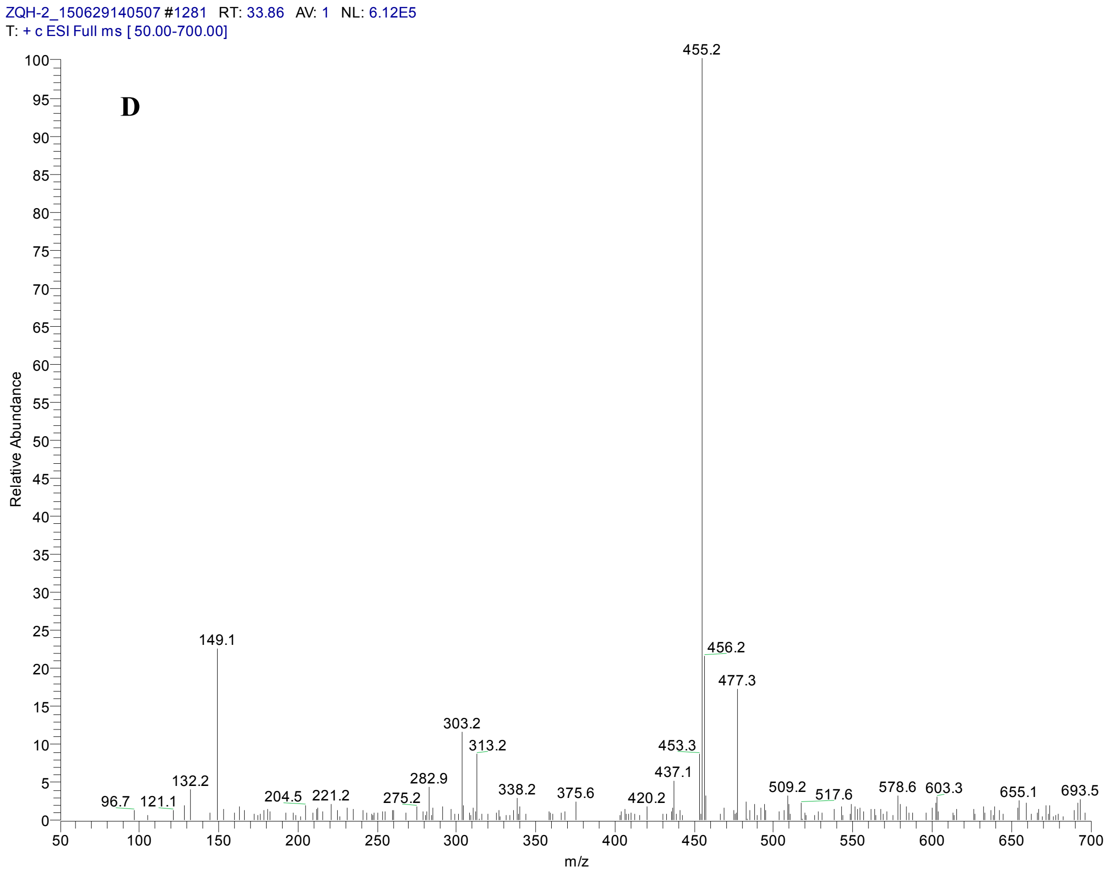
ZQH-2_150629140507 #1281 RT: 33.86 AV: 1 NL: 6.12E5
T: + c ESI Full ms [50.00-700.00]
D
Relative Abundance
m/z
455.2
456.2
477.3
149.1
303.2
313.2
453.3
437.1
132.2
96.7
121.1
204.5
221.2
275.2
282.9
338.2
375.6
420.2
509.2
517.6
578.6
603.3
655.1
693.5

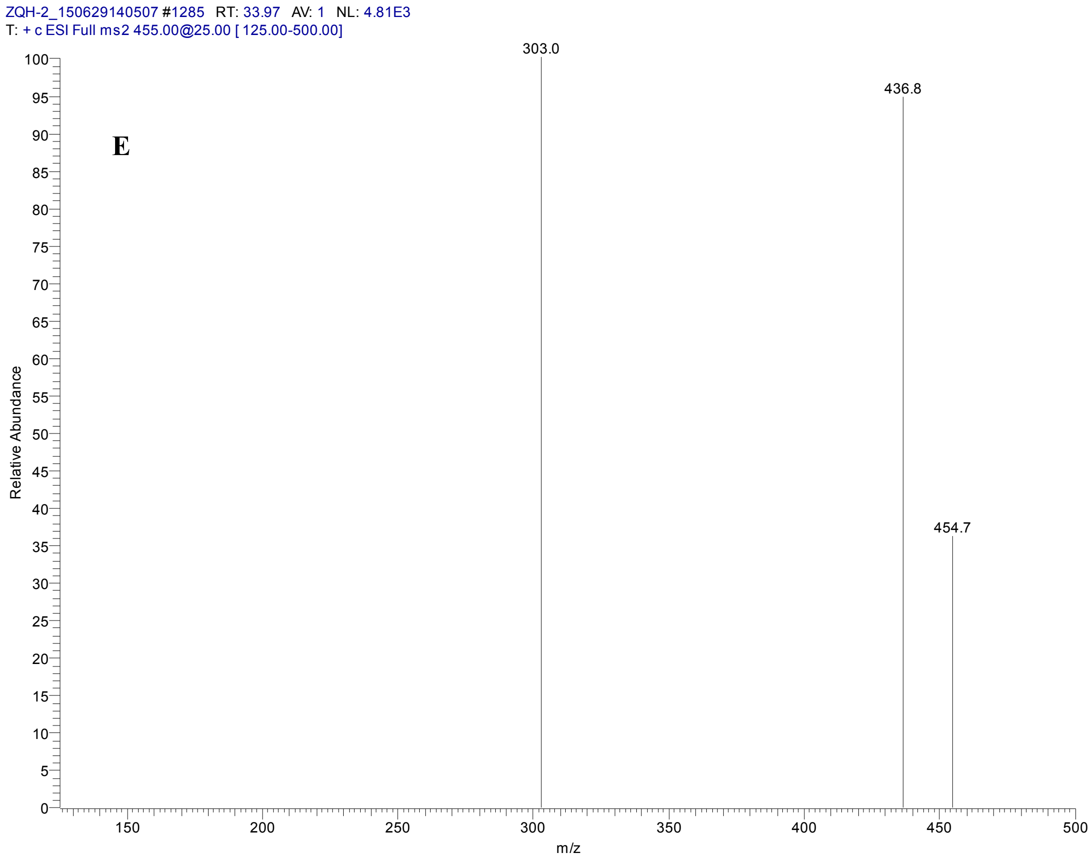
ZQH-2_150629140507 #1285 RT: 33.97 AV: 1 NL: 4.81E3
T: + c ESI Full ms2 455.00@25.00 [125.00-500.00]
E
303.0
436.8
454.7
Relative Abundance
m/z

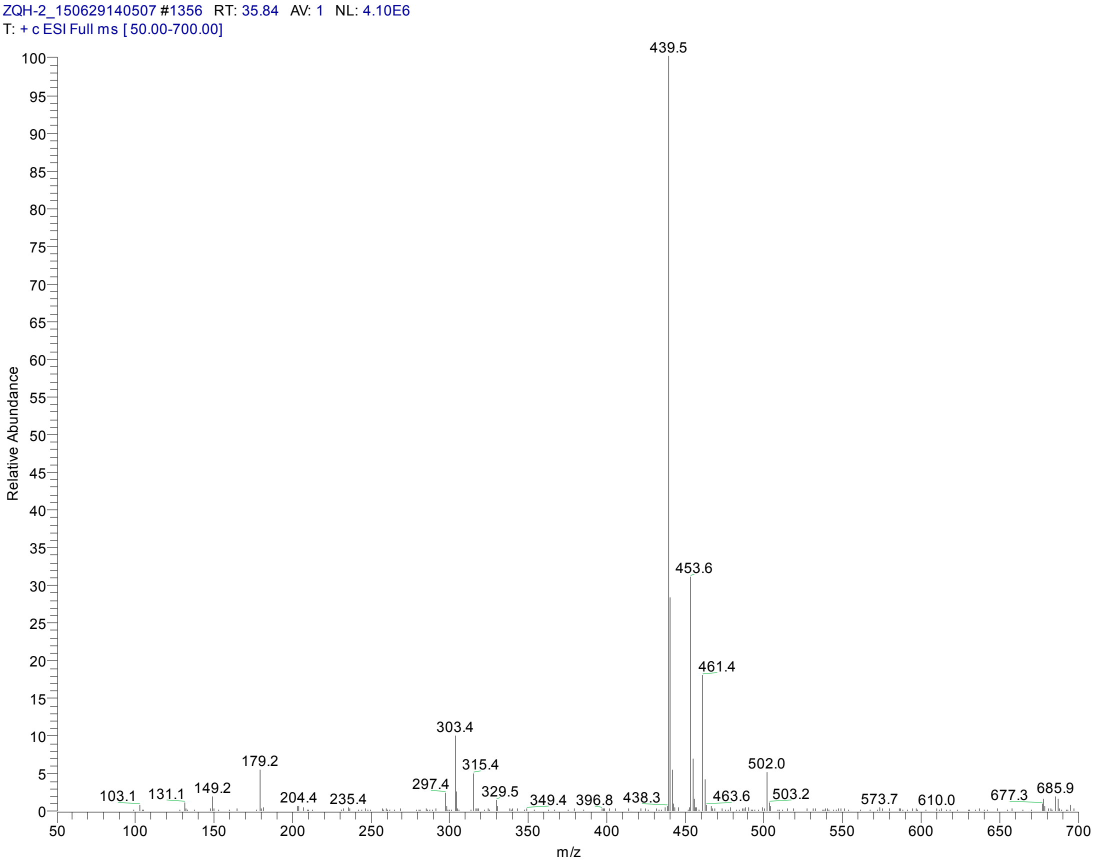
ZQH-2_150629140507 #1356 RT: 35.84 AV: 1 NL: 4.10E6
T: + c ESI Full ms [50.00-700.00]
439.5
453.6
461.4
303.4
179.2
315.4
502.0
297.4
329.5
149.2
103.1
131.1
204.4
235.4
349.4
396.8
438.3
463.6
503.2
573.7
610.0
677.3
685.9
Relative Abundance
m/z

F

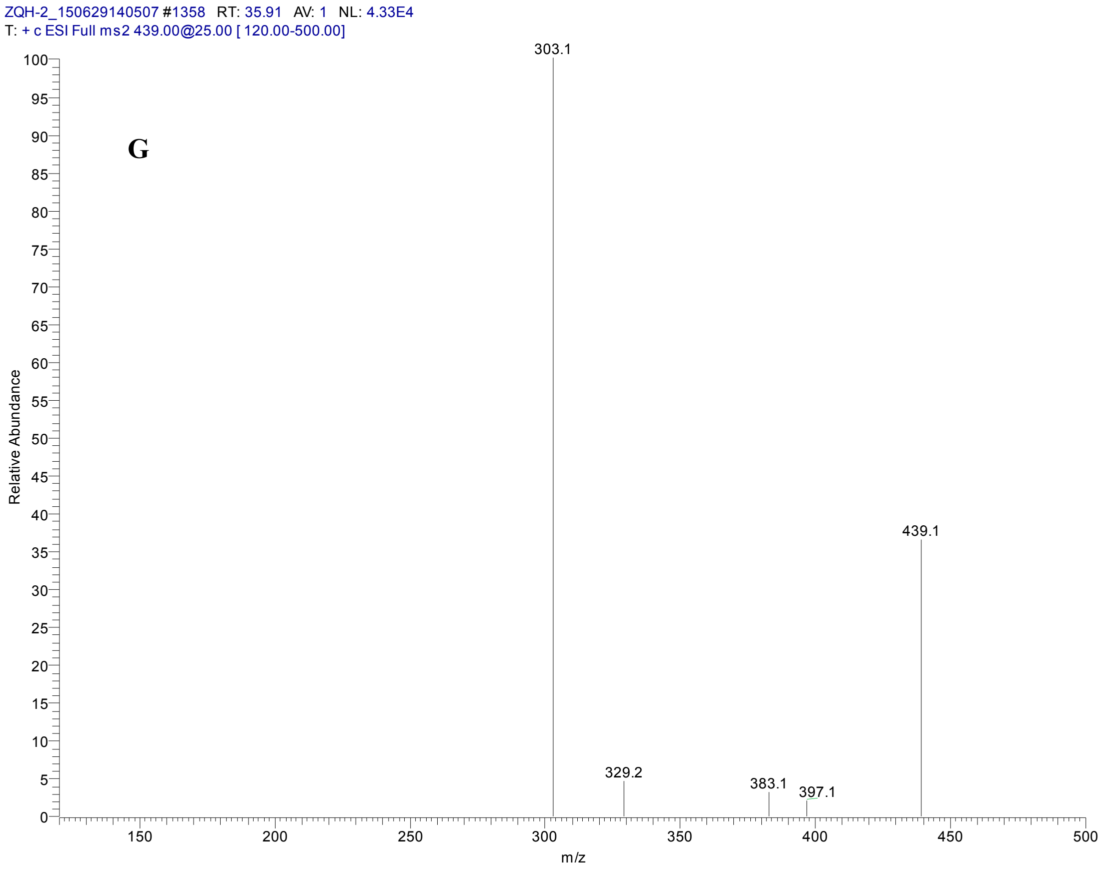

Figure S1. HPLC-MS/MS chromatograms of the main constituents of flavonoids. (**A**) Typical HPLC-MS chromatograms of flavonoids; (**B**) $[M + 1]^+$ of peak **4**; (**C**) MS^2 of peak **4**; (**D**) $[M + 1]^+$ of peak **5**; (**E**) MS^2 of peak **5**; (**F**) $[M + 1]^+$ of peak **6**; (**G**) MS^2 of peak **6**.
